# Editors’ Message – March 2025

**DOI:** 10.1016/j.ajpc.2025.100962

**Published:** 2025-03-17

**Authors:** Nathan D. Wong, Erin D. Michos

**Affiliations:** a Mary and Steve Wen Cardiovascular Division, University of California, Irvine School of Medicine, United States; b Division of Cardiology, Johns Hopkins University School of Medicine, United States

As we begin our sixth year of publication of the American Journal of Preventive Cardiology (AJPC) we continue to see substantial growth in the number of submissions which nearly tripled from calendar year 2023 with 134 submissions to 2024 with 361 submissions! This is testament to the increasing number of authors who have trusted their fine work to the Journal as well as the increasing relevance of the field of preventive cardiology. There has also been a substantial increase in usage of the journal with more 360,000 downloads and views in 2024. While the Journal is receiving submissions and usage globally, the majority of both submissions and usage derives from the United States.

To accommodate the increase in submissions, we continue to enlarge our editorial board and are always interested to hear from those who would like to review for the Journal, including early career people. Consequently, we consider good reviewers for future editorial board positions. We are happy to announce the appointment of new associate editors Dr. Dinesh Kalra from the University of Louisville and Dr. Anandita Kulkarni from Baylor Scott & White Heart Hospital in Plano, Texas. We remain grateful to the entire editorial board who have been crucial to the success of the Journal and of course to the growing cadre of authors who have entrusted their fine work to the Journal, as well as Elsevier and the American Society for Preventive Cardiology (ASPC) staff. Without the superb dedication and cooperation of all, the Journal would not be where it is today.

Our March 2025 issue is one of the largest to date with over 30 contributions. A key feature in this issue is an article highlighting the sessions at the highly successful 2024 ASPC Congress last summer. This was followed by a contemporary review of sleep and cardiovascular health. Some original contributions to note included a comparative study on increasing provider awareness of lipoprotein (a) [Lp(a)] testing for patients at risk for cardiovascular disease (CVD), a global disease burden analysis of cardiometabolic disease attributable to second-hand smoke exposure, and a study on Lp(a), c-reactive protein, homocysteine, and risk of incident CVD from the Multiethnic Study of Atherosclerosis. In addition, there were articles on CVD health and risks of atrial fibrillation and its prognosis, a systematic review on digital health interventions for optimizing postpartum cardiovascular health, a study examining achievement of guideline-based lipid goals among very high risk patients with atherosclerotic CVD and diabetes, and an analysis of the association of thoracic aortic calcium with incident CVD and all-cause mortality according to burden of coronary calcium. Of further interest were papers on coronary plaque characteristics quantified by artificial intelligence-enabled plaque analysis in a multiethnic sample, biomarkers of residual risk and all-cause mortality after acute coronary syndrome, and a study of premorbid predictors of death at initial presentation of coronary heart disease in the Women's Health Initiative. One particular article in this issue that received quite a bit of media attention was a large observational real-world study about unhealthy alcohol use and risk of coronary heart disease in young and middle-aged adults. There were more articles as well, and we encourage you to read the entire jam-packed issue.

We continue to invite you to contact us should you have any suggestions on how to further improve the *Journal* and maintain it as *the place to go* to publish latest research, state-of-the-art review and commentaries that continue to drive forward the rapidly expanding and increasingly recognized field of preventive cardiology.

Author agreement, separate title page, and manuscript without identifiers are not applicable for Editor's Messages.

## Author agreement

The authors Nathan D. Wong, PhD and Erin Michos MD have agreed to the submission of this MS.

## Disclosures

The authors do not report any relevant disclosures.

## Funding

No funding was received for the preparation of this manuscript.

## CRediT authorship contribution statement

**Nathan D. Wong:** Writing – original draft, Writing – review & editing. **Erin D. Michos:** Writing – review & editing.

## Declaration of competing interest

The authors declare that they have no known competing financial interests or personal relationships that could have appeared to influence the work reported in this paper.

